# Theoretical Investigation on the ESIPT Process and Detection Mechanism for Dual-Proton Type Fluorescent Probe

**DOI:** 10.3390/ijms23042132

**Published:** 2022-02-15

**Authors:** Yunjian Cao, Xiangrui Yu, Chaofan Sun, Jingang Cui

**Affiliations:** College of Science, Northeast Forestry University, Harbin 150040, China; yjcao@nefu.edu.cn (Y.C.); xryu@nefu.edu.cn (X.Y.)

**Keywords:** alkaline phosphatase, fluorescence probe, double proton transfer, time-dependent density functional theory

## Abstract

Recently, a new fluorescent probe AE-Phoswas reported to detect the activity of alkaline phosphatases (ALP) in different living cell lines. Here, we present an in-depth computational analysis of the mechanism and source of the fluorescence of the AE-Phos probe. There is an intermediate product (AE-OH-Phos) in the experiment as well as a different configuration of products that may emit fluorescence. It is essential to investigate the origin of fluorescence and the detection mechanism of the probe, which could help us eliminate the interference of other substances (including an intermediate product and possible isomers) on fluorescence during the experiment. According to the change of geometric parameters and Infrared spectra, we deduce that the dual intramolecular hydrogen bonds of salicylaldehyde azine (SA) were enhanced at the excited state, while AE-OH-Phos was attenuated. Considering the complex ESIPT behavior of the dual proton-type probe, the potential energy surfaces were further discussed. It can be concluded that the single proton transfer structure of SA (SA-SPT) is the most stable form. Both the concerted double proton transfer process and stepwise single proton transfer process of SA were forbidden. The fluorescence for SA was 438 nm, while that of SA-SPT was 521 nm, which agrees with the experimentally measured fluorescence wavelength (536 nm). The conclusion that single proton transfer occurs in SA is once again verified. In addition, the distribution of electron-hole and relative index was analyzed to investigate the intrinsic mechanism for the fluorescence quenching of the probe and the intermediate product. The identification of the origin of fluorescence sheds light on the design and use of dual-proton type fluorescent probes in the future.

## 1. Introduction

The excited-state intramolecular proton transfer process (ESIPT) is widely found in nature and is one of the most important processes in biology and chemistry [1,2,3]. This process is a photo-transfer isomerization, in which a proton is transferred to the adjacent N, O, S or other heteroatoms. The ESIPT molecules generally have a larger Stokes shift and could avoid self-absorption. The special photophysical property of ESIPT molecules has been widely studied by researchers working in the fields of materials, physics, chemistry, biology and medicine [4,5,6,7,8,9,10,11,12].

In recent years, fluorescent sensing technology has developed rapidly [13,14,15]. The excellent characteristics of fluorescence sensing have inspired explorations amongst research workers of fluorescence probes, which are widely used in the fields of biochemistry, medicine, environmental science, and industry as cogent tools to monitor molecular interactions. Considering that the ESIPT reactions tend to impose a great impact on the fluorescence emission spectra but cause almost no change to the absorption spectra, ESIPT has been considered as an ideal recognition mechanism to design ratiometric fluorescence probes, which possess a large Stokes shift. An increasing number of researchers have designed and synthesized ESIPT-based fluorescent probes for the selective detection of various anions, metal ions, and small organic molecules [16,17,18,19,20,21]. There are also many theoretical studies for the mechanistic elaboration of various fluorescence probes, as well as the design of novel probes [22,23,24,25,26,27,28,29,30,31,32]. Fluorescent probes for ALP detection generally feature phosphate as a recognition moiety, and after cleavage of the phosphate ester group, a phenolic hydroxyl group will be formed. In the case of ESIPT-based probes, the -OH moiety generated upon the cleavage of the phosphate will form an intramolecular hydrogen bond with another heteroatom, along which the proton-transferred reaction could occur. Before the cleavage, the ESIPT process is not possible.

**Scheme 1:**
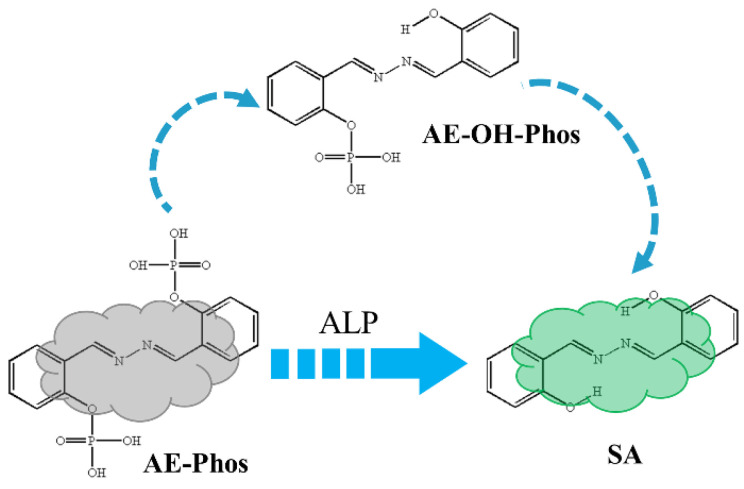
The experimental detection mechanism for ALP probe AE-Phos (Gray meaning no fluorescence, Green meaning green fluorescence).

Recently, Gao et al. [33] and Li [34] both designed a novel fluorescence probe for the detection of ALP activity and successfully applied it in living cells. He and colleagues also reported a new fluorescent probe, AE-Phos, for ALP activity detection, but the probe combined aggregation-induced emission and ESIPT and was able to image ALP well in different cell lines [35]. The detection mechanism of AE-Phos was shown in Scheme 1; the phosphate group was removed after AE-Phos reacted with ALP and the product SA and intermediate product AE-OH-Phos with ESIPT properties were generated. The authors suggested that the experimentally measured fluorescence spectra were emitted by SA. However, for such an ESIPT-based molecule, a single or double proton transfer process may happen at the excited state, which in turn emits fluorescence. In addition, the intermediate product may also emit fluorescence, so specific conclusions need to be studied in depth.

Herein, the methods of DFT and TD-DFT were used to investigate the probe AE-Phos and its possible products. In order to analyze the source of fluorescence and the possibility of single or double proton transfer processes occurring in SA, the potential energy surfaces for SA at the S_0_ and S_1_ states were scanned to ascertain their proton transfer process. Furthermore, the simulation of absorption and fluorescence spectra for the studied molecules not only verified the accuracy and reliability for the theoretical calculations, but also determined the concrete source of the experimental fluorescence. The causes of fluorescence quenching for the probe and its intermediate product were also investigated by electron-hole analysis. The possibility that all products and their corresponding isomers interfere with the fluorescence in the experimental process is excluded, and the specific source of fluorescence is determined, which will pave the way for experiments to understand and design better probe molecules.

## 2. Computational Details

All calculations in this work were performed employing Becke’s three-parameter hybrid exchange function with the Lee-Yang-Parr gradient-corrected correlation functional (B3LYP) and 6-311g(d,p) basis set [36,37,38,39,40] using density functional theory (DFT) [41,42,43] and time-dependent density functional theory (TD-DFT) [44,45,46,47,48] methods without any constraints under Gaussian 16 package [49]. To simulate the experimental environment, these calculations were accomplished in a Dimethyl sulfoxide (DMSO) solvent based on the integral equation formalism of the polarizable continuum model (IEFPCM). The geometrical structures of the ground and excited (S_0_ and S_1_) states for the probe and its products were separately optimized, and the relative geometric structure parameters were obtained. Furthermore, the infrared vibrations of the products were simulated based on the optimized structure, and the interaction region indicator (IRI) [50] isosurfaces combined with reduced density gradient (RDG) [51] scatter plots were plotted, applying Multiwfn 3.7 [52] and VMD [53] programs; all of these were used to analyze the change of hydrogen bond strength. The transition state theory is applied to calculate the energy change of excited-state isomerization. Additionally, the potential energy curves of AE-OH-PHOs were obtained by extending the distance of O_2_-H_2,_ and the surfaces of SA were scanned by regulating the distance of O_1_-H_1_ and O_2_-H_2_. In addition, the spectra were simulated, employing a B3PW91 functional.

## 3. Results and Discussion

### 3.1. Intramolecular Hydrogen Bond

**Figure 1:**
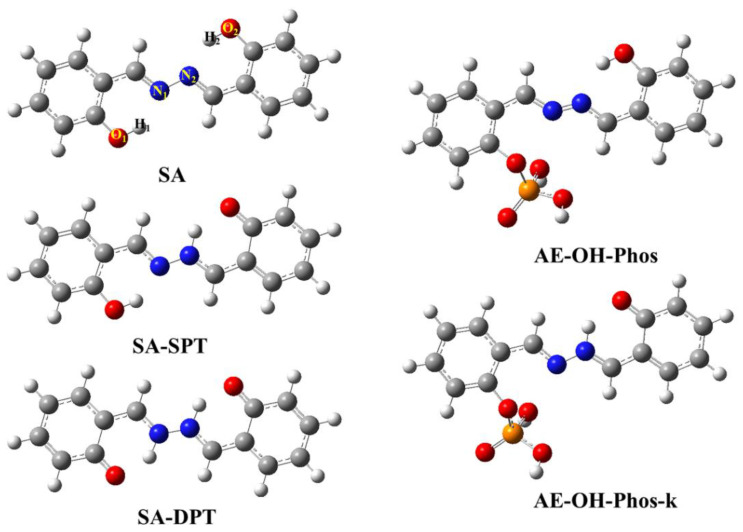
Possible structures of probe product SA and intermediate product AE-OH-Phos (SPT: single proton transfer; DPT: double proton transfer).

By analyzing the change in the strength of intramolecular hydrogen bonds (IHBs) upon photo-excitation, we can judge whether it is permissive for the ESIPT process and thus leads to isomerization. Therefore, the structures of all isomers for AE-OH-PHOs (enol and keto forms) and SA (named SA, SA-SPT, SA-DPT according to the proton transfer) have been optimized using the B3LYP/6-311G(d,p) basis set in the DMSO solvent, which is helpful to compare with the experiment. As shown in Figure 1, all structures of the probe products were included, and the atomic numbers associated with the hydrogen bonds were marked on the molecular structure of SA. The bond length and bond angle parameters related to molecules AE-OH-PHOs and SA were presented in Table 1 and Table 2, respectively.

In general, the longer the bond length (O-H distance) for the proton donor, the shorter the bond length (H…N distance) for the proton acceptor, and the closer the bond angle δ(O-H…N) is to 180°, the more stable the IHB [54,55,56]. For AE-OH-Phos, from the S_0_ state to S_1_ state (Table 1), the shortening of the O_2_-H_2_, the elongation of H_2_…N_2_, and the decrease of δ(O_2_-H_2_…N_2_) at the enol configuration indicate that the IHB is stronger and its structure is more stable at the S_0_ state, which is detrimental to the ESIPT reaction. In addition, the probe product SA had a completely symmetrical planar structure, with the same parameters for the two hydrogen bonds. Taking O_2_-H_2_…N_2_ as an example (Table 2), the O_2_-H_2_ length increased from 0.99 Å (S_0_) to 1.00 Å (S_1_), and the H_2_-N_2_ diminished from 1.76 Å to 1.70 Å, accompanied by the O_2_-H_2_-N_2_ angle, which enlarged from 146.1° to 148.3°. In addition, for SA-SPT that had undergone a single-step proton transfer process, it should be noted that the O_1_-H_1_, H_1_…N_1_ length and O_1_-H_1_…N_1_ angle were separately changed from 0.98 Å, 1.80 Å, and 144.7° (S_0_) to 0.99 Å, 1.77 Å, and 146.4° (S_1_), respectively. Although there is a trend of hydrogen bond enhancement, based on the slight changes of these structural parameters, we think it is not enough to support us to analyze whether the ESIPT process could occur. Therefore, the previously reported infrared spectra and the RDG scatter plots are used to further analyze the change of hydron bond strength. In addition, for O_2_…H_2_-N_2_ of which SA-SPT had undergone proton transfer, the change trend in the bond length and bond angle parameters indicates that the S_1_-state SA-SPT is more stable than the S_0_ state. However, the SA-DPT configuration is not stable in the S_0_ state. The detailed reasons will be discussed in the potential energy surfaces section.

**Figure 2:**
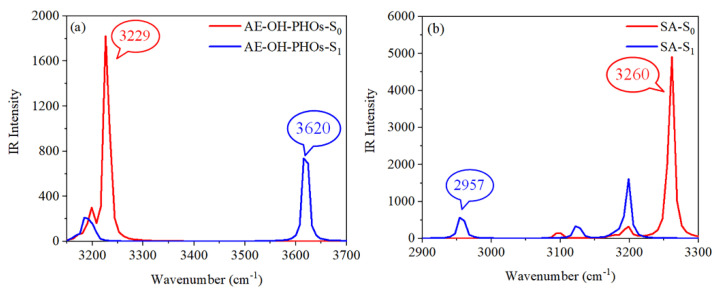
(**a**,**b**) IR spectra of probe product SA and intermediate product AE-OH-Phos.

Infrared (IR) spectra is a frequently used method to analyze the change of hydrogen bond; the degree of change for hydrogen bond strength can be judged by comparing the red-shift or blue-shift of hydroxyl stretching vibration peaks in the S_0_ and S_1_ states. The IR spectra for AE-OH-PHOs and SA are displayed in Figure 2. The calculated O_2_-H_2_ stretching vibration frequencies were separately located at 3229 cm^−1^ and 3620 cm^−1^ at the S_0_ and S_1_ states, respectively. Such a large blue-shift of 391 cm^−1^ demonstrates that the IHB at the S_1_ state is weaker than that at the S_0_ state, which is unfavorable for the ESIPT process. However, the stretching vibration frequencies of O_1_-H_1_ and O_2_-H_2_ for SA both change from 3260 cm^−1^ (S_0_) to 2957 cm^−1^ (S_1_), respectively, with a 303 cm^−1^ red-shift, which indicates that this is prone to ESIPT reaction. This result further validates our conjecture on the geometric structure.

**Figure 3:**
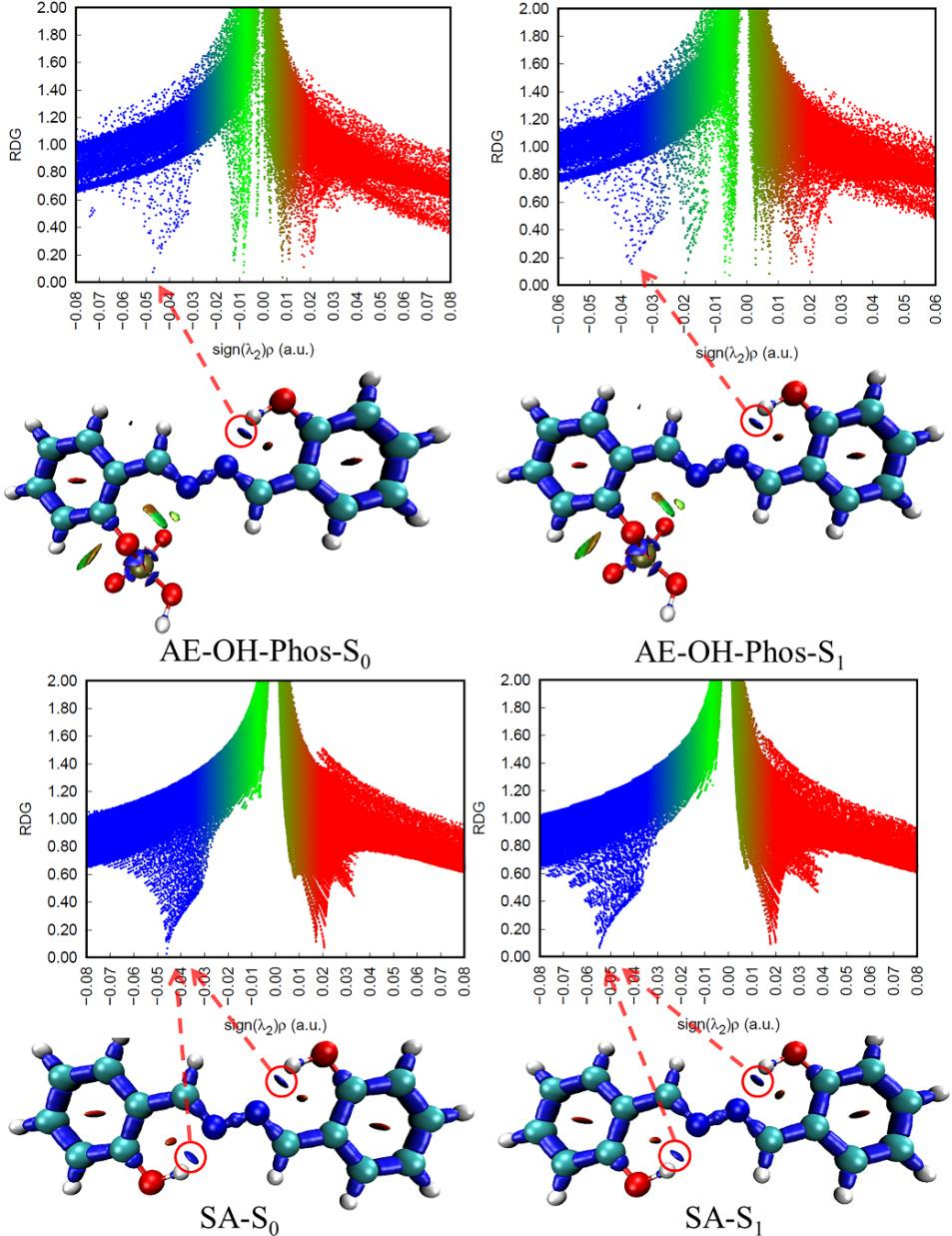
RDG scatter plots and IRI isosurfaces of product SA and AE-OH-Phos.

For the sake of more intuitively showing whether the IHB is formed and the change of IHB strength, the IRI isosurfaces and RDG scatter plots were subjected to further analysis. The meaning of different colors in Figure 3 are: red, green, and blue represent the steric effect, van der Waals interaction and hydrogen bond, respectively. Additionally, the bluer the IRI surface, the more negative the blue spike of RDG, indicating a stronger IHB. As shown in Figure 3, the appearance of a blue isosurface between H_2_ and N_2_ atoms for the intermediate product AE-OH-Phos at the S_0_ and S_1_ states signifies that IHB interaction exists and its corresponding RDG blue spike was separately near −0.05 a.u. and −0.04 a.u.. This change in value clearly shows that the IHB strength in the S_1_ state is weaker, which is in full agreement with the conclusions of the previous structure parameters and IR analysis. Therefore, it can be speculated that the ESIPT process cannot occur in AE-OH-Phos. Furthermore, SA, being a completely symmetric structure, has two IHBs of the same strength with the blue spikes of the S_0_ and S_1_ states located at −0.05 a.u. < ρ <−0.04 a.u. and −0.06 a.u. < ρ < −0.05 a.u.. The enhancement of IHB is very favorable for the ESIPT process after photoexcitation. However, whether the SA molecule underwent stepwise single proton transfer or concerted double proton transfer requires further analysis.

### 3.2. ESIPT Process Analysis

**Figure 4:**
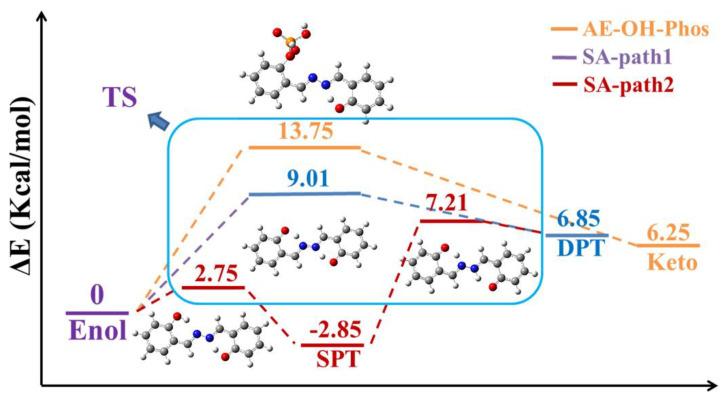
The transition state structure and energy of SA and AE-OH-Phos.

To calculate the precise reaction energy barrier for the proton transfer process occurring in SA and AE-OH-Phos at the S_1_ state, the transition state (TS) structure of its different paths and the corresponding energies were calculated. The TS structure and energy for SA stepwise single proton transfer (path 1) and simultaneous double proton transfer (path 2), as well as AE-OH-Phos, are shown in Figure 4. The energy barrier of AE-OH-Phos is up to 13.75 kcal/mol; this indicates that the proton transfer process was forbidden at the S_1_ state. In addition, the reaction barrier of single proton transfer is 2.75 kcal/mol and the double proton transfer is 9.01 kcal/mol. This suggests that the SA is more inclined to undergo single proton transfer into the SA-SPT form than the process of double proton transfer. Moreover, the SA-SPT has the lowest energy, which means that its configuration is the most stable.

**Figure 5:**
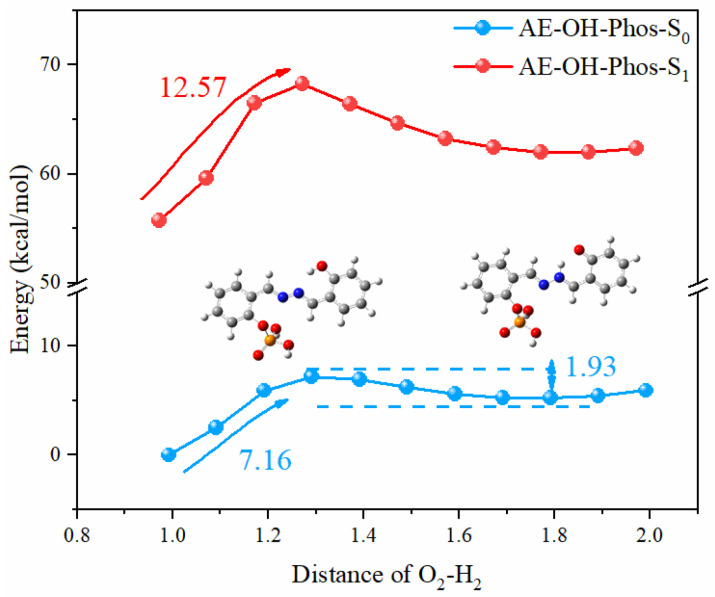
The potential energy curves of AE-OH-Phos.

To be able to investigate the intrinsic mechanism of the ESIPT process and the possible proton transfer for AE-OH-Phos and SA, the potential energy surfaces (PESs) of SA and the potential energy curves (PECs) of AE-OH-Phos at the S_0_ and S_1_ states were scanned separately based on the optimized structure. The obtained PECs of AE-OH-Phos were as a function of O_2_-H_2_ distance and ranged from 0.99 Å(initial length)→1.99 Å and 0.97 Å(initial length)→1.97 Å at the S_0_ and S_1_ states, respectively. As depicted in Figure 5, the reverse potential barrier of 1.93 kcal/mol is lower than the forward potential barrier (7.16 kcal/mol), indicating that it is difficult for the proton transfer process to occur at the S_0_ state. This is consistent with the previously obtained result that weakening of the S_1_-state hydrogen bond is unfavorable for the ESIPT reaction to proceed.

**Figure 6:**
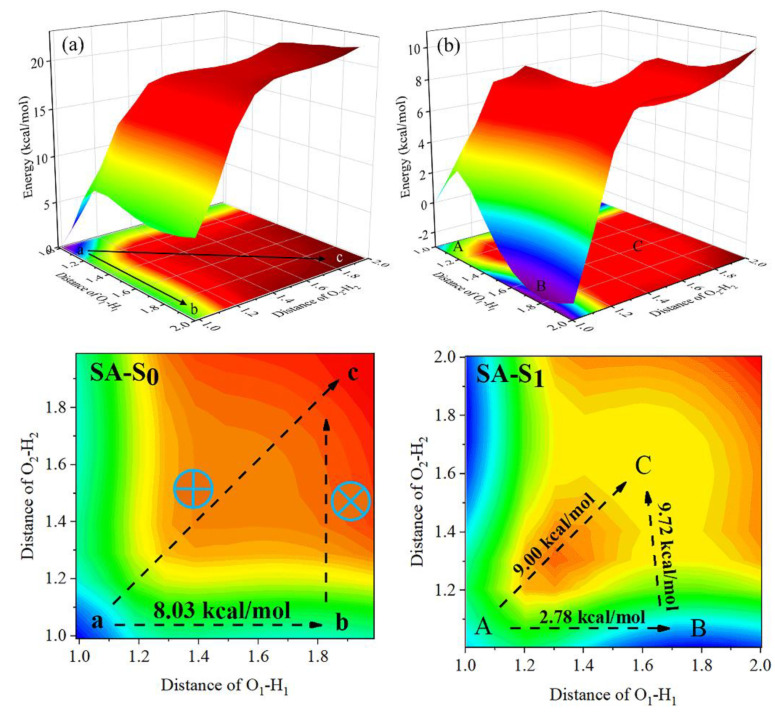
The potential energy surfaces and contours of SA at (**a**) S_0_- and (**b**) S_1_- state.

The PESs of SA were obtained by extending the distance of O_1_-H_1_ and O_2_-H_2_, as shown in Figure 6. It is worth noting that the PESs of both the S_0_ and S_1_ states are symmetric, and the lowest energy points in the figures are marked for the convenience of analysis. In Figure 6a, the coordinates of these corresponding points are a (0.99 Å, 0.99 Å), b (1.99 Å, 0.99 Å), and c (1.99 Å, 1.99 Å). In addition, the energy of all points on the S_0_-state PES is higher than that of point a; this means that the structure of point a is the most stable at the S_0_ state. Furthermore, the potential energy of paths a→c and b→c increases monotonically, which indicates that these two paths do not actually exist at the S_0_ state. Moreover, the forward energy barrier of a→c is 8.03 kcal/mol, while the reverse energy barrier is only 0.31 kcal/mol, so it can be concluded that the isomerization process cannot happen at the S_0_ state either.

Interestingly, the calculated potential energies of the minima points are in order of E_C_ > E_A_ > E_B_ at the S_1_ state, as present in Figure 6b. The coordinates of these corresponding points are A (1.00 Å, 1.00 Å), B (1.80 Å, 1.00 Å) and C (1.60 Å, 1.60 Å). It is clear that point B is the most stable configuration at the S_1_ state, and the path from A to B only needs to cross the energy barrier of 2.78 kcal/mol, which means that the S_1_-state SA molecule could easily undergo a single proton transfer process to form SA-SPT. Nevertheless, the energy barrier for the path A→C to cause a concerted double proton transfer process is 9.00 kcal/mol, and even though this energy barrier is not very high, the barrier of A→B is significantly lower than it, which demonstrates that the synchronous double proton transfer for SA is forbidden at the S_1_ state. After reaching point B, the SA-SPT needs to cross the potential barrier of 9.72 kcal/mol to reach point C. To analyze whether the proton transfer process in the second step can occur, the reverse proton transfer energy barrier for all paths was calculated: C→B, C→A, and B→A are 0.12 kcal/mol, 2.11 kcal/mol, and 5.49 kcal/mol, respectively. The results prove that even if the proton transfer from B to C occurred, it would return to point B with an almost no-barrier process. It can be concluded that the SA-SPT conformation is stable to exist at point B. Based on the analysis of the ESIPT behavior of the intermediate product AE-OH-Phos and product SA, the following conclusions can be drawn: due to the presence of phosphate group AE-OH-Phos, which is unable to experience ESIPT process, SA can neither undergo stepwise nor synergistic double proton transfer, but can only proceed with a single proton transfer process, which is stable in the form of SA-SPT.

### 3.3. Electronic Spectra and Fluorescence Mechanism

Absorption and fluorescence spectra are often used to fit theoretical calculations to experiments to verify the reliability and accuracy of a theory. Therefore, the spectra of ALP probe product SA were simulated separately, employing different functions in a DMSO solvent of IEFPCM at 6-311G(d,p) basis set, as listed in Table 3. The previous analysis can determine that the single proton transfer process of SA is definitely present, and the simulated fluorescence spectra are also the closest to the experimental value of SA-SPT. Hence, the Stokes-shift of SA-SPT was calculated using different functionals and found that the value (145 nm) of B3PW91 is the closest to the experimental value (180 nm). Therefore, the spectra of studied molecules were simulated by B3PW91 for subsequent discussion and the first three single excited state transition and fluorescence properties are separately gathered in Table 4 and Table 5.

**Figure 7:**
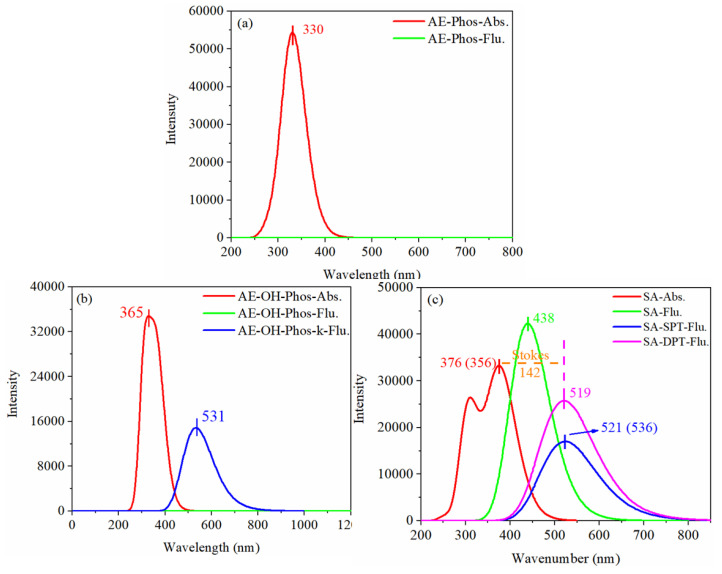
The absorption and fluorescence spectra (**a**) probe AE-Phos, (**b**) intermediate product AE-OH-phos, (**c**) product SA (Experimental values are in parentheses).

To compare the spectral changes more visually, their spectra are plotted in Figure 7. It can be found that the fluorescence of probe AE-Phos and intermediate product AE-OH-Phos was quenched, which means that neither fluorescence can be observed experimentally. Moreover, the result is consistent with the experimentally measured spectra. As listed in Table 4, the oscillator strength of S_1_-state AE-Phos was 0.0010, which means that the fluorescence is almost unobservable experimentally. That is to say; the S_1_ state is a dark state. The electrons of AE-Phos cannot be directly excited to the S_1_ state, but are excited to the S_2_ state. According to Kasha’s rule, the electrons of the S_2_ state will return to the S_1_ state through internal conversion, resulting in fluorescence quenching. However, the electrons of AE-OH-Phos were excited to the S_1_ state but a fluorescence quenching also occurred, which we speculate may be due to the photo-induced electron transfer process; this will be further analyzed in the frontier molecular orbitals section. More importantly, the PEC shows that AE-OH-Phos is unable to undergo the ESIPT process into the keto form. As a consequence, we can conclude that AE-OH-Phos does not interfere with the experimental spectra by emitting fluorescence.

As shown in Figure 7c, the two peaks of the absorption band of SA are consistent with the experimentally measured peaks. The highest peak in the absorption band at 376 nm is produced by the electron transitions to the S_1_ state, which is in line with the experimental value (356 nm), and combined with Table 4, we can know that another peak at a smaller wavelength is emitted from the S_0_ state transitions to the S_4_ state. In addition, the calculated fluorescence peak of SA-SPT (521 nm) is also in line with the experiment (536 nm), which is consistent with the conclusion that the SA-SPT form is stable the S_1_ state obtained from our previous analysis. In a word, the probe AE-Phos produces the product SA when detecting the ALP activity, and the intermediate product AE-OH-PHOs may also be present, but since both AE-Phos and AE-OH-PHOs are fluorescence quenching, they will not affect the fluorescence spectra of the experiment. Moreover, the fluorescence of SA-SPT is almost identical to the experimental fluorescence; it is presumed that the fluorescence measured in the experiment is derived from SA-SPT, which is formed by SA undergoing a single proton transfer process.

### 3.4. Frontier Molecular Orbitals

**Figure 8:**
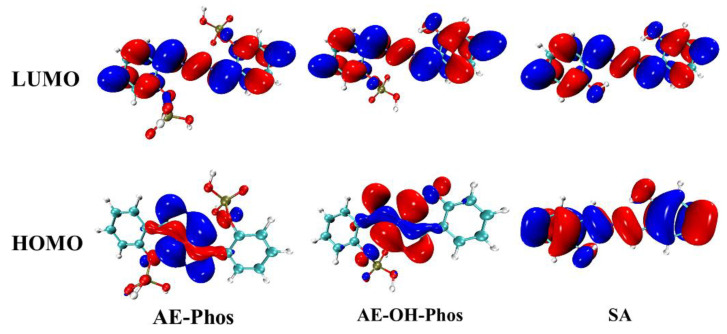
Frontier molecular orbitals of ALP probe and its products.

In the previous section, we mentioned that the fluorescence of AE-PHOs and AE-OH-PHOs is quenched, and this change in photophysical properties is attributed to the redistribution of electrons due to photoexcitation. To observe the change in charge distribution after photoexcitation, the highest occupied molecular orbital (HOMO) and the lowest unoccupied molecular orbital (LUMO) corresponding to their fluorescence emission are plotted in Figure 8. When the electrons of AE-PHOs and AE-OH-PHOs fluoresce from LUMO to HOMO, there is an obvious concentration for charge from the benzene ring on both sides to the middle near the N-N bond. Such a charge transfer process could cause the chromophore part to fail to emit fluorescence, and this process is called the photo-induced electron transfer process. However, SA has almost no significant change in electron cloud distribution due to local excitation.

**Figure 9:**
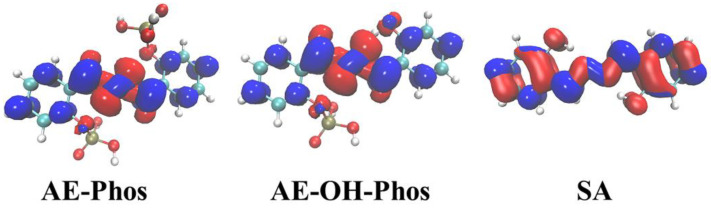
The electrons (blue) and holes (red) distribution for the studied molecules.

In order to better visualize the distribution of electrons and holes, and thus discuss the intrinsic cause of the fluorescence quenching for the probe and its intermediate products, the electrons and holes distribution of three molecules was investigated using the electron-hole analysis method. The relevant parameters were gathered in Table 6. The D, *t*, H, and Δσ index reflect the distance between the center of mass for electrons and holes, the degree of separation, the overall average distribution breadth, and the difference between the overall spatial distribution breadth, respectively. Generally speaking, *t* > 0 and *t* < 0 separately indicate whether the charge transfer makes the electron-hole sufficiently separated. Combined with Table 6 and Figure 9, the *t* index of all three molecules is less than zero, but the figure shows an obvious separation of the electron-hole. In other words, the *t* index is not perfect enough to analyze multidirectional charge transfer. The Sr index means the degree of electron-hole overlap; the larger the value, the higher the degree of overlap. The smaller hole and electron delocalization index (HDI and EDI) illustrates the higher delocalization degree of the electron-hole, that is, the greater the distribution and uniformity.

As depicted in Figure 9, for AE-PHOs and AE-OH-PHOs, the holes are concentrated in the connecting part of the two benzene rings and the electrons are uniformly distributed throughout the framework of the molecule. The apparent movement of electrons from the middle part to the sides causes the fluorescence quenching in both molecules. The D index of the three molecules shows that the distance between their centers of mass is very short, again proving the opinion that the electrons are uniformly distributed on both sides. The Δσ index manifests that the electron distribution breadth for AE-PHOs and AE-OH-PHOs is larger than that of the holes, while SA is almost the same. The HDI of AE-PHOs and AE-OH-PHOs are approximately twice as high as the EDI, indicating that the electron delocalization of both molecules is greater than that of the holes. The electron-hole analysis verifies that AE-PHOs and AE-OH-PHOs produce a significant charge transfer, which is likely to lead to fluorescence quenching.

## 4. Conclusions

On the whole, theoretical calculations were performed to investigate the detection mechanism of the ALP probe and to analyze the origin of the fluorescence measured from the experiments. Geometric structure parameters, IR vibration, and RDG scatter plots indicate that the excited state IHB for SA is enhanced, while AE-OH-Phos is weakened. Combining the simulated spectra and potential energy curves (surfaces), AE-OH-Phos is unable to emit fluorescence, and the experimentally measured fluorescence is derived from the SA-SPT configuration formed by SA undergoing single proton transfer. The agreement between the simulated SA spectra and experimental spectra proves the accuracy and feasibility of the theoretical calculation. Finally, the distribution of frontier molecular orbital electron clouds and electron-hole analysis elucidates the endogenous causes of the fluorescence quenching for probe AE-Phos and intermediate product AE-OH-Phos. Our work based on the salicylaldehyde azine shows that the dual-proton type structure cannot undergo a double proton transfer process, but can only undergo a single proton transfer process. Additionally, the ESIPT-type intermediate AE-OH-Phos cannot in fact produce the ESIPT reaction. All of these could provide a theoretical reference for the designing and synthesizing of novel double proton transfer fluorescent probes.

## Figures and Tables

**Table 1:** Calculated relative bond lengths (Å) and bond angles (°) of the enol and keto forms for AE-OH-PHOs at the S_0_ and S_1_ states.

	State	O_2_-H_2_	H_2_-N_2_	δ(O_2_-H_2_-N_2_)
AE-OH-PHOs-e	S_0_	0.99	1.76	146.5
	S_1_	0.97	1.90	143.0
AE-OH-PHOs-k	S_0_	1.76	1.04	135.2
	S_1_	1.82	1.04	136.0

**Table 2:** Calculated relative bond lengths (Å) and bond angles (°) of SA, SA-SPT, and SA-DPT at the S_0_ and S_1_ states.

	SA		SA-SPT		SA-DPT	
State	S_0_	S_1_	S_0_	S_1_	S_0_	S_1_
O_1_-H_1_	0.99	1.00	0.98	0.99	—	1.64
H_1_-N_1_	1.76	1.70	1.80	1.77	—	1.06
O_2_-H_2_	0.99	1.00	1.73	1.79	—	1.64
H_2_-N_2_	1.76	1.70	1.05	1.04	—	1.06
δ(O_1_-H_1_-N_1_)	146.1	148.3	144.7	146.4	—	140.9
δ(O_2_-H_2_-N_2_)	146.1	148.3	136.3	137.0	—	140.8

**Table 3:** Performed functional correction by comparing the Stokes-shifts (nm) of the probe products SA.

	B3PW91	CAM-B3LYP	M06-2W	MPWPW91	WB97XD	Exp. ^a^
SA-Abs.	376	335	332	365	332	356
SA-Flu.	438	403	400	428	400	536
SA-SPT-Flu.	521	452	456	503	445	
SA-DPT-Flu.	519	461	468	504	456	
Stokes-shifts	145	117	124	138	113	180

^a^ Represents the maximum absorption peak measured in the experiment.

**Table 4:** The electron-excited properties of the probe and its products in DMSO solvent using the TD-DFT/B3PW91/6-311G(d,p) method.

	State	E (eV)	λ_abs_ (nm)	Contribution MO	*f*
AE-PHOs	S_1_	3.1819	390	(0.69920) H-1→L	0.0010
	S_2_	3.7584	330	(0.70193) H→L	1.3290
	S_3_	4.2759	290	(0.67557) H-2→L	0.0000
AE-OH-PHOs	S_1_	3.3963	365	(0.69704) H→L	0.6683
	S_2_	3.4735	357	(0.70157) H-2→L	0.0011
	S_3_	3.9535	314	(0.69131) H-1→L	0.6960
SA	S_1_	3.2932	376	(0.70025) H→L	0.7985
	S_2_	3.6328	341	(0.69900) H-1→L	0.0000
	S_3_	3.7808	328	(0.70259) H-3→L	0.0003
	S_4_	4.0515	306	(0.69207) H-2→L	0.6351

**Table 5:** The fluorescence properties of the probe and its products in DMSO solvent using the TD-DFT/B3PW91/6-311G(d,p) method.

	State	E (eV)	λ_flu_ (nm)	Contribution MO	*f*
AE-PHOs	S1	0.6158	2013	(0.70484) H→L	0.0000
AE-OH-PHOs	S1	1.1122	1115	(0.70423) H→L	0.0000
AE-OH-PHOs-k	S1	2.3363	531	(0.70719) H→L	0.3710
SA	S1	2.8331	438	(0.70031) H→L	1.0443
SA-SPT	S1	2.3784	521	(0.70706) H→L	0.4243
SA-DPT	S1	2.3882	519	(0.70758) H→L	0.6405

**Table 6:** Indexes related to the distribution of electrons and holes for the probe and its products.

	D (Å)	*t* (Å)	H (Å)	Δσ (Å)	Sr (a.u.)	HDI	EDI
AE -PHOs	0.010	−1.879	2.477	1.750	0.375	16.49	8.23
AE-OH-PHOs	0.303	−1.537	2.504	1.696	0.381	16.48	8.20
SA	0.000	−3.382	3.698	−0.167	0.755	6.78	7.49

## Data Availability

Not applicable.
